# “Emerging Food Processing and Novel Approaches for Extraction and Application of Bioactive Compounds”: Special Issue Editorial Overview

**DOI:** 10.3390/molecules28083523

**Published:** 2023-04-17

**Authors:** Mohsen Gavahian, Changwei Hsieh

**Affiliations:** 1 Department of Food Science, National Pingtung University of Science and Technology, 1, Shuefu Road, Neipu, Pingtung 91201, Taiwan; 2 Department of Food Science and Biotechnology, National Chung Hsing University, 145 Xingda Rd., South Dist., Taichung 40227, Taiwan

The guest editors Mohsen Gavahian and Changwei Hsieh are pleased to present the editorial overview of the Special Issue entitled “Emerging Food Processing and Novel Approaches for Extraction and Application of Bioactive Compounds”. This Special Issue addressed recent trends in emerging food processing technologies and innovative approaches for extracting and applying bioactive compounds in the food, pharmaceutical, and cosmetics industries.

Naturally occurring bioactive compounds, such as polyphenols, phytosterols, fatty acids, flavonoids, caffeine, carotenoids, and essential oils, have been widely recognized due to 21st century consumer demands for healthy and functional foods. However, due to the long processing times involved, conventional extraction and processing technologies have several limitations, such as low efficiency and the degradation of bioactive compounds. In light of this, researchers have been exploring emerging food processing technologies and innovative approaches for extracting and applying bioactive compounds, including phytochemicals, to address these challenges.

The aforementioned Special Issue attracted both researchers and pioneers in the field, with a total number of 12 submissions from authors affiliated with institutions from various countries around the world, including Azerbaijan, Iran, Poland, Russia, Spain, Sudan, Taiwan, Turkey, and the United States. The acceptance rate was 50%, including desk and peer review rejections.

The articles included in the Special Issue cover a broad range of topics and provide valuable insights into the latest research updates in the field. For example, Mostashari et al. discussed the impact of processing and extraction methods on the allergenicity of targeted proteins and bioactive peptides derived from eggs [1]. This high-quality review work explained the present challenges and perspectives to maximize product functionality while reducing allergenicity. Another study provided information on the “Effect of D-Limonene Nanoemulsion Edible Film on Banana (*Musa sapientum* Linn.) Post-Harvest Preservation” [2]. The researchers in this study showed how bio-based coating could provide anti-bacterial activity and air/moisture barrier properties to extend bananas’ shelf life. Yang et al. elaborated on enhancing the bioactive saponin content of Raphanus sativus extract by utilizing thermal processing under various conditions [3]. This article provided evidence that treatment could induce functional saponin conversion in plants to improve the health efficacy of plant-based products.

Another research team reported on enhancing the anti-oxidant property of N-Carboxymethyl Chitosan and its application in strawberry preservation [4]. The input of these researchers provides valuable information on an emerging food packing method based on bioactive compounds, which was proven effective for strawberries. Eltayeb et al. offered helpful information on the essential oils composition and biological activity of *Chamaecyparis obtusa*, *Chrysopogon nigritanus*, and *Lavandula coronopifolia* grown in the wild in Sudan [5]. The results reported by these authors revealed that their essential oils have significant anti-acetylcholinesterase and anti-butyrylcholinesterase activities. The study also provided evidence that the essential oils of *C. obtusa, C. nigritanus,* and *L. coronopifolia* are potential natural additives with good functional properties in the food, pharmaceutical, and cosmetics industries. Lastly, one manuscript focused on the high-pressure homogenization of red beetroot juice [6]. This innovative study assessed the physicochemical properties and betalain pigments that were affected by this process. The paper provided evidence to show that betaxanthins and vulgaxanthins I and II resist high-pressure homogenization. It was also revealed in the study that betanin had the lowest degradation rate compared to other betacyanins. The new information provided in this study helps to contribute to food processing and dietary-supplement-producing industries as betanin is the primary betalain pigment, a natural food colorant with health-promoting bioactive effects.

In conclusion, the aforementioned Special Issue provides an informative overview of the latest research updates on emerging food processing techniques and novel approaches for the extraction and application of bioactive compounds. The guest editors of this Special Issue hope that it will inspire future studies in the field and help to develop innovative strategies to address the challenges faced by the food, pharmaceutical, and cosmetics industries.
